# Engagement of the EP_2_ prostanoid receptor closes the K^+^ channel K_Ca_3.1 in human lung mast cells and attenuates their migration

**DOI:** 10.1002/eji.200738106

**Published:** 2008-09

**Authors:** S Mark Duffy, Glenn Cruse, Sarah L Cockerill, Chris E Brightling, Peter Bradding

**Affiliations:** The Department of Infection, Immunity and Inflammation, Institute for Lung Health, University of LeicesterUK

**Keywords:** Chemotaxis, Ion channel, K_Ca_3.1, Mast cell, Prostaglandin E_2_

## Abstract

Human lung mast cells (HLMC) express the Ca^2+^-activated K^+^ channel K_Ca_3.1, which plays a crucial role in their migration to a variety of diverse chemotactic stimuli. K_Ca_3.1 activation is attenuated by the β_2_-adrenoceptor and the adenosine A_2A_ receptor through a G_s_-coupled mechanism independent of cyclic AMP. Prostaglandin E_2_ promotes degranulation and migration of mouse bone marrow-derived mast cells through the G_i_-coupled EP_3_ prostanoid receptor, and induces LTC_4_ and cytokine secretion from human cord blood-derived mast cells. However, PGE_2_ binding to the G_s_-coupled EP_2_ receptor on HLMC inhibits their degranulation. We show that EP_2_ receptor engagement closes K_Ca_3.1 in HLMC. The EP_2_ receptor-specific agonist butaprost was more potent than PGE_2_ in this respect, and the effects of both agonists were reversed by the EP_2_ receptor antagonist AH6809. Butaprost markedly inhibited HLMC migration induced by chemokine-rich airway smooth muscle-conditioned media. Interestingly, PGE_2_ alone was chemotactic for HLMC at high concentrations (1 µM), but was a more potent chemoattractant for HLMC following EP_2_ receptor blockade. Therefore, the G_s_-coupled EP_2_ receptor closes K_Ca_3.1 in HLMC and attenuates both chemokine- and PGE_2_-dependent HLMC migration. EP_2_ receptor agonists with K_Ca_3.1 modulating function may be useful for the treatment of mast cell-mediated disease.

## Introduction

Mast cells are tissue-dwelling cells derived from bone marrow progenitors. They are present in all organs throughout the human body, both at mucosal surfaces and within connective tissues. Mast cells play a major role in tissue homeostasis, host defence and the pathophysiology of many diverse diseases 1. These include pulmonary fibrosis, rheumatoid disease and atherosclerosis, but they are most commonly associated with allergic disease due to their activation by allergen 2. In many diseases mast cells re-locate to specific compartments within tissue. This is typified in asthma where mast cells migrate into the airway epithelium 3, airway smooth muscle (ASM) 4 and submucosal glands 5. This places activated mast cells in direct contact with these dysfunctional airway elements, allowing the specific delivery of detrimental cell–cell signals. Drugs that inhibit this tissue relocation by preventing mast cell migration may prove particularly effective in the treatment of mast cell-mediated disease.

Human mast cells express the intermediate conductance Ca^2+^-activated K^+^ channel K_Ca_3.1, which plays a critical role in their migration to diverse chemotactic stimuli 6, and to a lesser extent in their degranulation 7, 8. Drugs that directly block this channel or which close it indirectly therefore have potential as novel therapies for mast cell-dependent disease. K_Ca_3.1 in human lung mast cells (HLMC) is closed by both salbutamol and adenosine *via* the β_2_-adrenoceptor and A_2A_ adenosine receptors, respectively 9, 10, which are both G_s_-coupled G protein-coupled receptors (GPCR). In keeping with a critical role for K_Ca_3.1 in HLMC migration, adenosine also inhibits HLMC chemotaxis *via* the A_2A_ receptor 10.

PGE_2_ is a prostanoid with four specific GPCR, designated EP_1–4_. EP_2_ and EP_4_ couple to G_s_, EP_3_ couples predominantly to G_i_ although two isoforms also couple to G_s_, while EP_1_ receptors mobilise intracellular Ca^2+^, probably through G_q_ 11. PGE_2_ therefore has diverse biological activities depending on the receptors it interacts with and the cells expressing them. With respect to mast cells, mouse mast cells express the EP_3_ receptor whose activation induces both degranulation and chemotaxis 12, 13. Human cord blood-derived mast cells, express both EP_2_ and EP_3_ receptors. In these cells, PGE_2_ enhances degranulation and PGD_2_ production in cells primed by IL-4, an effect mediated through the EP_3_ receptor. However, following FcɛRI-dependent activation PGE_2_ inhibits human cord blood-derived mast cell LTC_4_, PGD_2_, IL-5 and TNF-α production *via* the EP_2_ receptor but has no effect on degranulation 14. In contrast, several studies have shown that PGE_2_ consistently inhibits degranulation and eicosanoid production by HLMC, an effect mediated *via* the EP_2_ receptor 15–17. This receptor also appears to dominate PGE_2_-dependent signalling on mast cells within the human asthmatic lung as PGE_2_ markedly attenuates both the early and late phase airway response to allergen challenge 18.

Since K_Ca_3.1 in HLMC is closed by G_s_-coupled β_2_-adrenoceptors and A_2A_ adenosine receptors, we hypothesised that activation of the G_s_-coupled EP_2_ receptor would also close K_Ca_3.1 in these cells. Furthermore, if PGE_2_ was to close K_Ca_3.1, then it should inhibit HLMC chemotaxis. To test this hypothesis we have used the patch-clamp technique to investigate the effects of PGE_2_ on HLMC ion channel function, and investigated the effect of PGE_2_ on HLMC migration induced by asthmatic ASM-conditioned medium.

## Results

### PGE_2_ alone does not open K_Ca_3.1

We initially examined whether PGE_2_ opens K_Ca_3.1 in HLMC under resting baseline conditions. HLMC are typically electrically silent at rest and it is possible that PGE_2_ could open either K_Ca_3.1 or another channel as reported previously for adenosine 10. No significant change in current amplitude was observed in seven cells following the addition of 10^−5^ M PGE_2_ (current changed from 1.22±0.54 to 1.42±0.85 pA at +40 mV; *p*=0.678).

### PGE_2_ closes K_Ca_3.1 in the presence of the K_Ca_3.1 opener 1-EBIO

We then examined whether PGE_2_ closes K_Ca_3.1. Because PGE_2_ might potentially inhibit many IgE-dependent cell activation pathways that could reduce cytosolic-free Ca^2+^, and thus reduce K_Ca_3.1 activity indirectly, we concentrated on studying the effects of PGE_2_ on K_Ca_3.1 currents that were induced by the K_Ca_3.1 opener 1-EBIO 8–10. This compound opens K_Ca_3.1 with a half-maximal value of about 30 µM for heterologously expressed K_Ca_3.1, with a maximal effect at about 300 µM 19. 1-EBIO is specific for K_Ca_3.1 in HLMC and opens it by enhancing the channels sensitivity to [Ca^2+^]_i_ 19. Thus at 100 µM EBIO, maximal K^+^ currents are achieved in the presence of 100 nM free Ca^2+^, which is below the resting [Ca^2+^]_i_ of most cell types including HLMC 8.

**Figure 1:**
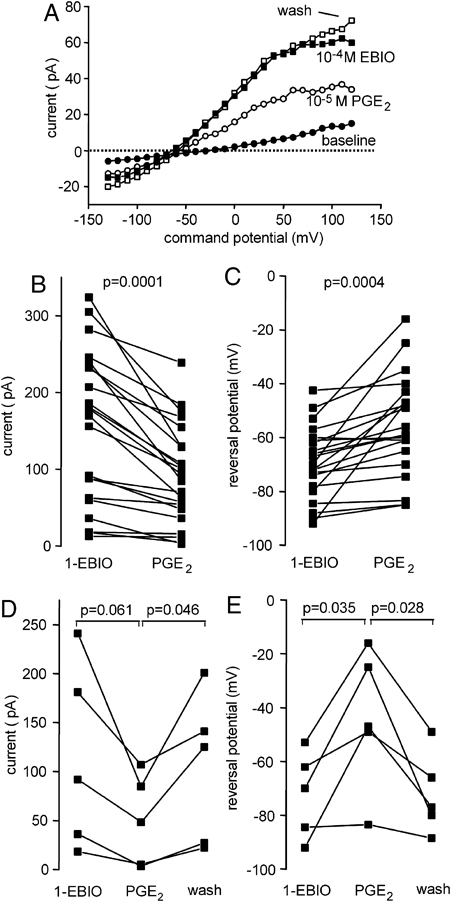
PGE_2_ closes K_Ca_3.1 in HLMC in a consistent and reversible manner. (A) Current–voltage curve demonstrating suppression of a 1-EBIO-induced K_Ca_3.1 current by 10^−5^ M PGE_2_ and partial reversal of the effect following removal of PGE_2_ (wash). (B) Suppression of K_Ca_3.1 current measured at +40 mV by 10^−5^ M PGE_2_ (*n*=22 cells). (C) Shift in whole-cell current reversal potential (Vm) by 10^−5^ M PGE_2_ (*n*=22 cells). (D) K_Ca_3.1 current measured at +40 mV after addition of 1-EBIO, suppression by 10^−5^ M PGE_2_ and then reversibility of suppression following removal of PGE_2_ (wash) (*n*=5 cells). (E) Whole-cell current reversal potential (Vm) after the addition of 1-EBIO, a depolarising positive shift in response to 10^−5^ M PGE_2_, and then reversibility following removal of PGE_2_ (wash) (*n*=5 cells).

In cells in which K_Ca_3.1 had been activated by 1-EBIO, addition of PGE_2_ (10^−8^–10^−5^ M) produced a rapid (within 30 s) dose-responsive inhibition of channel activity with an associated positive shift in membrane potential (Fig. 1A–E). PGE_2_ 10^−5^ M suppressed the K_Ca_3.1 current in >90% of cells (Fig. 1B). Thus, addition of 10^−5^ M PGE_2_ reduced K_Ca_3.1 membrane current at +40 mV from 155.4±20.9 to 92.6±13.7 pA (*p*=0.0001, *n*=22 cells)(Fig. 1B), with a corresponding shift in reversal potential (Vm) from −69.5±2.8 to −56.3±3.9 mV (*p*=0.0004) (Fig. 1C). Half maximal suppression (IC_50_) of K_Ca_3.1 by PGE_2_ occurred at approximately 4.0×10^−7^ M (calculated from six cells). Importantly, the effect of PGE_2_ was partially reversed within 1 min by removing it from the recording solution (current post PGE_2_ 49.6±20.8 pA, post wash 103.4±34.5 pA, *p*=0.046; Vm post PGE_2_ −44.1±11.7 mV, post wash −72.1±6.81 mV, *p*=0.028, *n*=5) (Fig. 1D and E), indicating that non-specific “rundown” was not responsible for the effects seen.

### K_Ca_3.1 modulation by PGE_2_ is mediated *via* EP_2_ receptors

**Figure 2:**
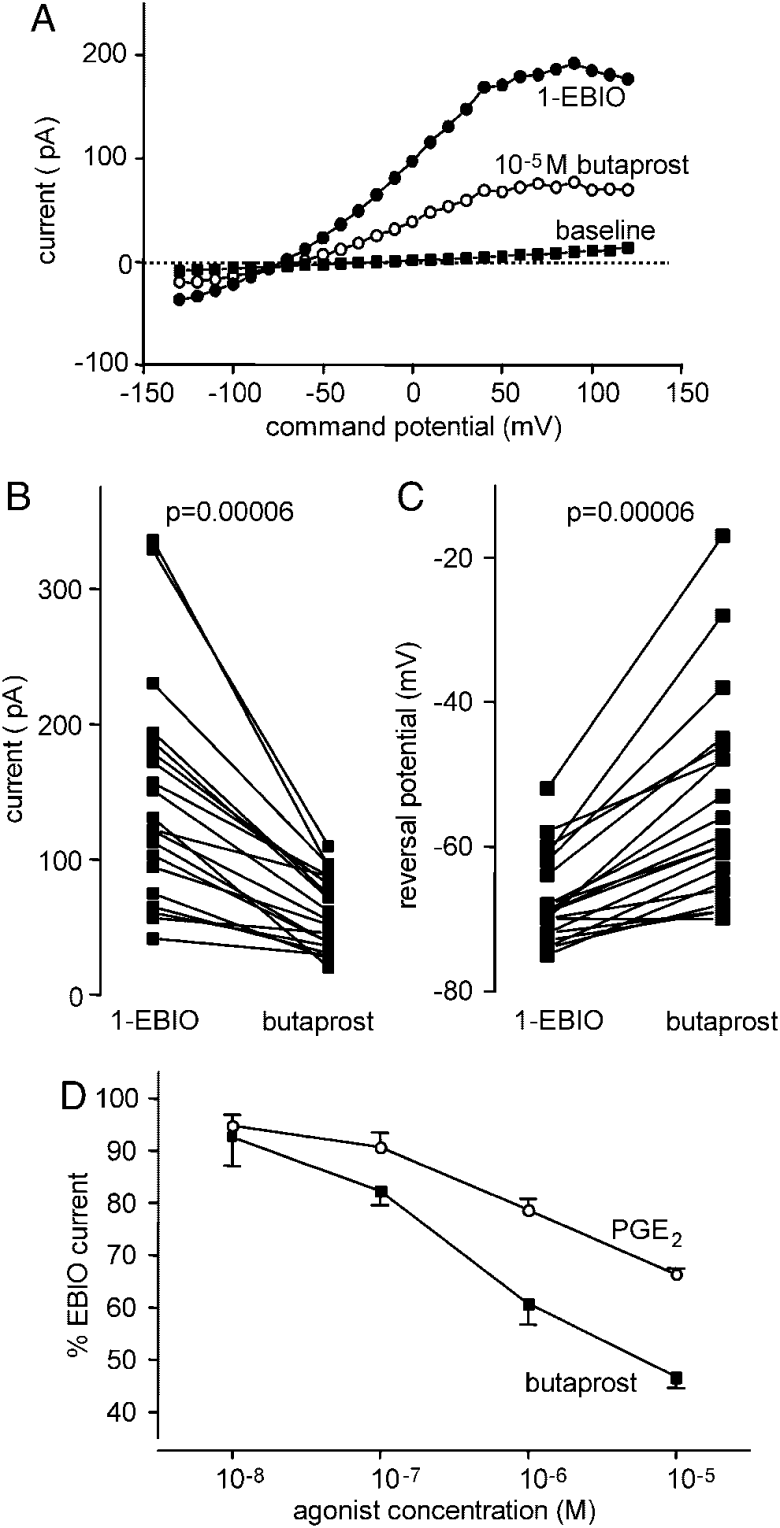
The EP_2_ receptor agonist butaprost closes K_Ca_3.1 in HLMC. (A) Current–voltage curve demonstrating suppression of a 1-EBIO-induced K_Ca_3.1 current by 10^−5^ butaprost. (B) Suppression of K_Ca_3.1 current measured at +40 mV by 10^−5^ M butaprost (*n*=20 cells). (C) Shift in whole-cell current reversal potential (Vm) by 10^−5^ M butaprost (*n*=20 cells). (D) Concentration–response curves for K_Ca_3.1 suppression by PGE_2_ and the EP_2_ agonist butaprost (mean±SEM of five cells for each).

To examine whether the effects of PGE_2_ were mediated via EP_2_ prostanoid receptors we examined the effects of EP_2_ receptor agonists/antagonists. First, we examined the effects of the selective EP_2_ receptor agonist butaprost. Butaprost mimicked the effects of native PGE_2_ in a dose-dependent manner (Fig. 2A–D). At a concentration of 10^−5^ M, butaprost reduced the K_Ca_3.1 current from 146.0±18.3 pA to 61.2±6.1 pA (*p*=0.00006, *n*=20) (Fig. 2B) with a corresponding shift in reversal from −67.5±1.4 to −54.4±3.2 mV (*p*=0.00006) (Fig. 2C). Half maximal suppression (IC_50_) of K_Ca_3.1 by butaprost occurred at approximately 2.1×10^−7^ M (*n*=6 cells) (Fig. 2D).

**Figure 3:**
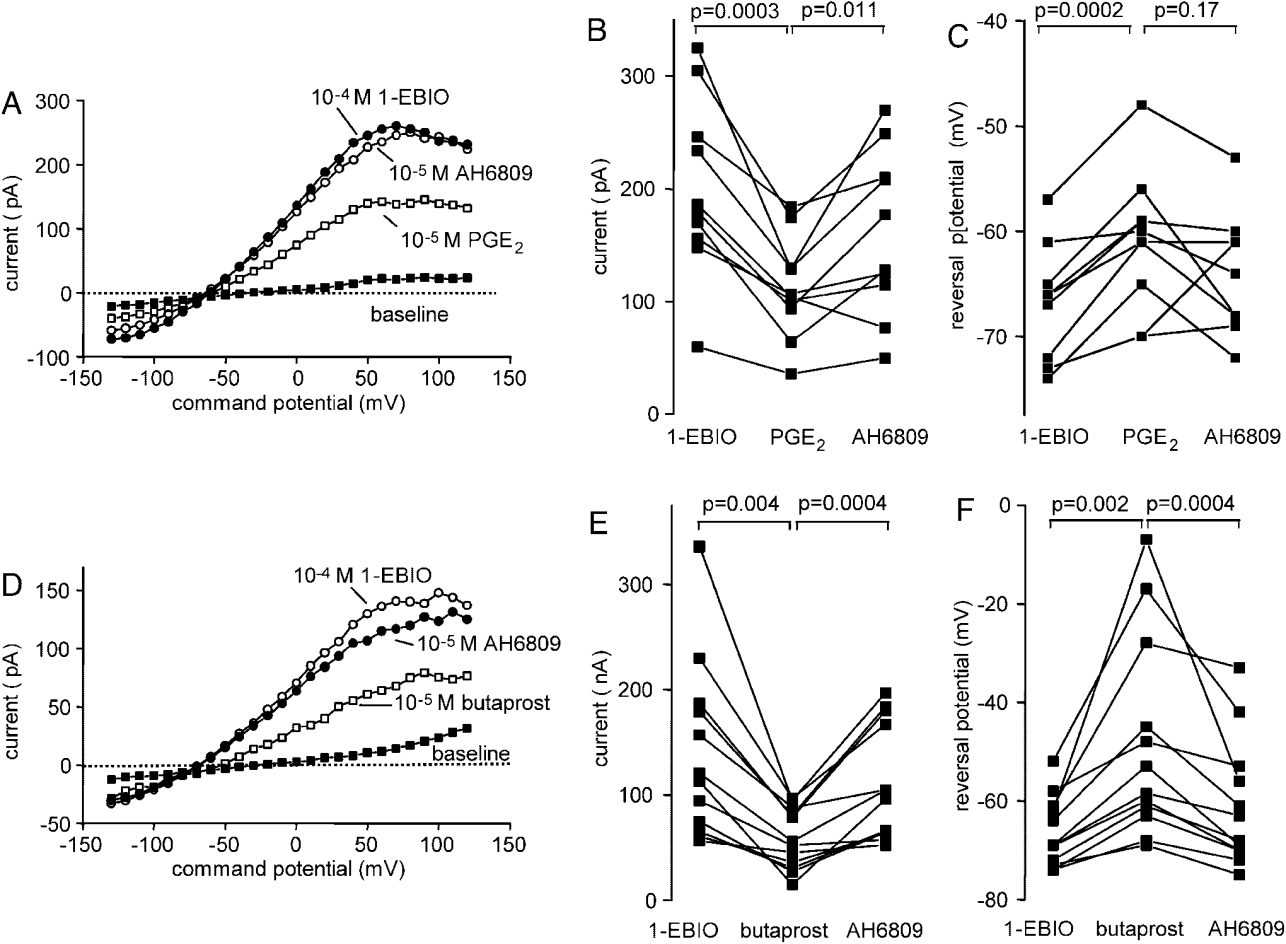
The effect of the EP_1_/_2_ receptor antagonist AH6809 on PGE_2_- and butaprost -dependent closure of K_Ca_3.1. (A) Current–voltage curve demonstrating reversibility of K_Ca_3.1 suppression by PGE_2_ following administration of the EP_1/2_ receptor antagonist AH6809. (B) K_Ca_3.1 current measured at +40 mV after addition of 1-EBIO, suppression by 10^−5^ M PGE_2_ and then reversibility of suppression following addition of AH6809 (*n*=10 cells). (C) Whole-cell current reversal potential (Vm) after the addition of 1-EBIO, a depolarising positive shift in response to 10^−5^ M PGE_2_, and then following addition AH6809 (*n*=10 cells). (D) Current–voltage curve demonstrating reversibility of K_Ca_3.1 suppression by butaprost following administration of AH6809. (E) K_Ca_3.1 current measured at +40 mV after addition of 1-EBIO, suppression by 10^−5^ M butaprost and then reversibility of suppression following addition of AH6809 (*n*=12 cells). (F) Whole-cell current reversal potential (Vm) after the addition of 1-EBIO, a depolarising positive shift in response to 10^−5^ M PGE_2_, and then reversibility following addition AH6809 (*n*=12 cells).

**Figure 4:**
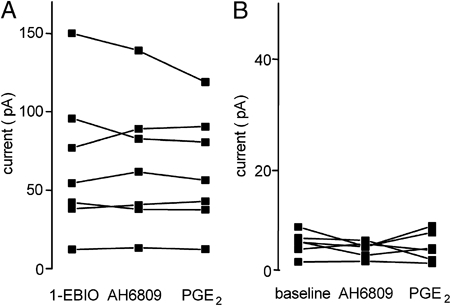
PGE_2_ is ineffective in the presence of the AH6809. (A) K_Ca_3.1 current measured at +40 mV after addition of 1-EBIO, stability following addition of AH6809, and failure to suppress following subsequent addition of 10^−5^ M PGE_2_. (B) K_Ca_3.1 current measured at +40 mV in resting cells, after addition of AH6809, and then following subsequent addition of 10^−5^ M PGE_2_ showing failure of K_Ca_3.1 to open.

The suppression of K_Ca_3.1 by 10^−5^ M PGE_2_ was partially reversed by the competitive EP_1_ and EP_2_ receptor antagonist AH6809 (Fig. 3A–C). Thus, in experiments studying AH6809 at a concentration of 10^−5^ M, current at +40 mV was 112±14.3 pA post PGE_2_, increasing to 160.9±23.2 pA post AH6809 (*p*=0.011, *n*=10 cells) (Fig. 3B). There was however no significant shift in reversal potential in these experiments explained by the fact that significant K_Ca_3.1 currents remained following PGE_2_ application (Vm post PGE_2_ −60.9±2.1 mV, post AH6809 −63.6±1.8 mV, *p*=0.17) (Fig. 3C). We also examined the effect of AH6809 on the selective EP_2_ agonist butaprost. The suppression of K_Ca_3.1 by 10^−5^ M butaprost was also partially reversed by 10^−5^ M AH6809 (Fig. 3D–F). Current at +40 mV was 58.5±8.3 pA post butaprost, increasing to 111.6±15.9 pA post AH6809 (*p*=0.0004, *n*=12) (Fig. 3E). There was also a significant shift in reversal potential (Vm post butaprost −48.1±5.8 mV, post AH6809 −61.0±3.7 mV, *p*=0.0004) (Fig. 3F). In the presence of AH6809, PGE_2_ had no effect on K_Ca_3.1 currents that had been induced by 1-EBIO (current at +40 mV 67.0±17.2 pA post 1-EBIO, 66.2±15.7 post AH6809, 62.7±13.7 pA post PGE_2_, *n*=7)(Fig. 4A). Similarly, K_Ca_3.1 currents did not appear in resting cells to which AH6809 was added prior to PGE_2_ (current 6.9±0.9 pA at baseline, 5.8±0.6 pA post AH6809, 6.3±1.1 pA post PGE_2_, *n*=6) (Fig. 4B). The EP_1_ and EP_3_ receptor agonist 17-phenyl trinor PGE_2_ did not close K_Ca_3.1 after activation with 1-EBIO (current at +40 mV post 1-EBIO 128.3±94.9 pA, post 17-phenyl trinor PGE_2_ 127.8±90.4 pA, *p*=0.9567, *n*=6) and did not open K_Ca_3.1 in resting cells (baseline current at +40 mV 5.70±0.88 pA, current post 17-phenyl trinor PGE_2_ 5.36±0.949 pA, *p*=0.202, *n*=5). These results exclude a role for EP_1_, EP_3_ or EP_4_ receptors in K_Ca_3.1 modulation. Taking the data together, it can be concluded that suppression of K_Ca_3.1 by PGE_2_ is mediated by the EP_2_ receptor.

### PGE_2_ closes K_Ca_3.1 following IgE-dependent activation

**Figure 5:**
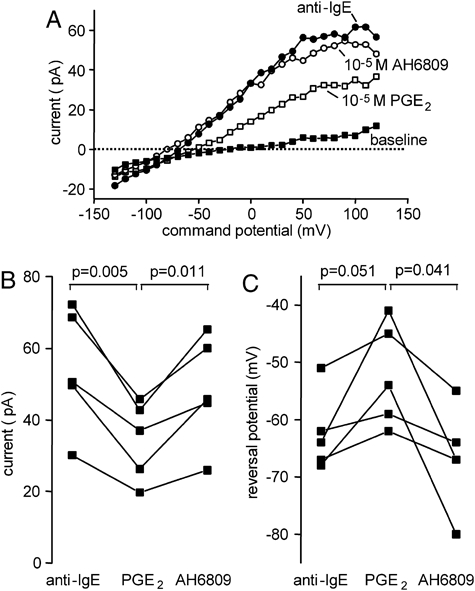
The effect of 10^−5^ M PGE_2_ on K_Ca_3.1 currents elicited by anti-IgE-dependent mast cell activation. (A) A representative HLMC demonstrating the development of a K_Ca_3.1 current following anti-IgE-dependent activation, suppression of this by 10^−5^ M PGE_2_, and reversal of the PGE_2_-induced suppression by the EP_1/2_ receptor antagonist AH6809. (B) K_Ca_3.1 current measured at +40 mV after addition of anti-IgE, suppression by 10^−5^ M PGE_2_ and then reversibility of suppression following addition of AH6809 (*n*=5 cells). (C) Whole-cell current reversal potential (Vm) after the addition of anti-IgE, a depolarising positive shift in response to 10^−5^ M PGE_2_, and then reversibility following addition AH6809 (*n*=5 cells).

Lastly, we confirmed whether PGE_2_-dependent regulation of K_Ca_3.1 was relevant to K_Ca_3.1 channels that had been opened by anti-IgE-dependent activation. Anti-IgE (1:1000 dilution) opened K_Ca_3.1 in 5/5 cells tested (baseline current at +40 mV 5.8±1.3 pA, baseline Vm −17.8±3.2 mV; post anti-IgE current 54.2±7.6 pA, Vm −62.4±3.0 mV). PGE_2_ (10^−5^ M) suppressed the current to 34.3±4.9 pA post PGE_2_, *p*=0.005) (Fig. 5A and B) and produced an associated positive shift in Vm to −52.2±4.1 mV, *p*=0.051) (Fig. 5C). This suppressive effect of PGE_2_ was partially reversed by AH6809 (current 48.3±6.9 pA, *p*=0.011; Vm −66.6±4.0 mV, *p*=0.041) (Fig. 5A and C).

### PGE_2_ suppresses HLMC migration through the EP_2_ receptor

**Figure 6:**
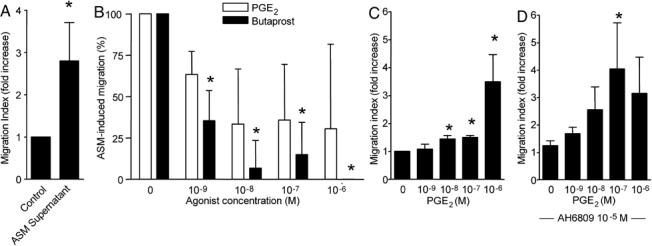
Inhibition of HLMC chemotaxis through EP_2_ receptor activation. (A) HLMC migration using conditioned medium from asthmatic airway smooth muscle as the chemotactic stimulus. (B) HLMC migration is attenuated significantly by butaprost (n=4) but not PGE_2_ (*n*=3) in the presence of ASM-conditioned medium. ^*^p<0.05 compared with control (no PGE_2_ or butaprost). (C) PGE_2_ is chemotactic when present in isolation. ^*^p<0.05 compared to control (no PGE_2_), *n*=4. (D) PGE_2_-dependent chemotaxis is enhanced in the presence of the EP_1/2_ receptor antagonist AH6809 (10^−5^ M). ^*^p<0.05 compared to control (no AH6809), *n*=4.

Conditioned medium from asthmatic ASM, which has been activated with TNFα, IFNγ and IL-1β, mediates HLMC chemotaxis predominantly *via* the CXCL10/CXCR3 pathway with additional contributions from ligands for CXCR1 and CXCR3 20. Inhibition of K_Ca_3.1 by channel blockers markedly suppresses this HLMC chemotaxis 6, as do molecules that close K_Ca_3.1 such as adenosine 10. Migration of HLMC using conditioned medium from asthmatic airway smooth muscle was 2.8±0.9 fold that of medium control (*n*=4, *p*=0.046) (Fig. 6A) and this was not inhibited significantly by PGE_2_ (Fig. 6B). However, the selective EP_2_ agonist butaprost produced marked inhibition of HLMC migration to ASM-conditioned medium (Fig. 6B). Interestingly, PGE_2_ was chemotactic on its own, but much less potent than in mouse bone marrow-derived mast cells 13 (Fig. 6C). This HLMC chemotactic activity of PGE_2_ was markedly increased in the presence of EP_1/2_ blockade by AH6809 10^−5^ M (Fig. 6D). HLMC migration to both ASM-conditioned medium and PGE_2_ itself is therefore attenuated by the EP_2_ receptor.

### PGE_2_ attenuates histamine release from cultured HLMC

PGE_2_ inhibits the degranulation of HLMC freshly isolated from lung tissue. Because the cells used in this study were cultured in stem cell factor (SCF), IL-6 and IL-10, we investigated whether PGE_2_ also inhibits release from these HLMC. In keeping with observations in freshly isolated HLMC, PGE_2_ inhibited histamine release dose dependently over the concentration range 10^−9^–10^−6^ M (control mean±s.e.m. net histamine release 19.8±3.7% versus 7.5±3.3% with 10^−6^ PGE_2_, *n*=4 donors, *p*=0.046).

### K_Ca_3.1 is not modulated by G_i_ or G_q_-coupled receptors

We have now identified three distinct G_s_-coupled receptors that close K_Ca_3.1 in HLMC when exposed to the relevant ligands, and in the case of the β_2_-adrenoceptor, the inverse agonist ICI 118551 opens the channel 9. In contrast, the G_i_-coupled CXCR3 receptor does not couple directly to K_Ca_3.1 6. We have therefore investigated further G_i_ and G_q_ agonists known to have biological effects on HLMC. Platelet activating factor (PAF, 10^−7^ M) (G_i_), lysophosphatidic acid (LPA, 10^−5^ M) (G_q,12/13,i_) and UTP (10^−4^ M)(G_q_) did not open K_Ca_3.1 in resting HLMC (*n*=≥6 cells for each agonist) and did not close K_Ca_3.1 that had been opened by 1-EBIO (*n*=≥5 cells for each agonist)(data not shown).

## Discussion

In this study we have examined the effects of EP_2_ receptor activation on K_Ca_3.1 ion channel function in HLMC. In keeping with the known effects of the β_2_-adrenoceptor and A_2A_ adenosine receptor 9, 10, the G_s_-coupled EP_2_ receptor also closes this channel reversibly. Consistent with this effect on the channel, EP_2_ receptor activation attenuates HLMC migration, and in consequence masks the potential chemotactic activity of PGE_2_ on HLMC, which is particularly evident when the EP_2_ receptor is blocked pharmacologically.

PGE_2_ closed K_Ca_3.1 reversibly following IgE-dependent mast cell activation demonstrating physiological relevance. PGE_2_ also closed K_Ca_3.1 channels that had been activated by the K_Ca_3.1 opener 1-EBIO. The inhibition of K_Ca_3.1 by PGE_2_ was reversed both by removing it from the recording solution, and by the addition of the competitive EP_1_ and EP_2_ receptor antagonist AH6809 indicating a receptor-mediated mechanism. Since the effects of PGE_2_ were mimicked by the specific EP_2_ receptor agonist butaprost but not the EP_1_ agonist 17-phenyl trinor PGE_2_, and no effects of native PGE_2_ were seen in the presence of the EP_1/2_ receptor antagonist AH6809, we have firm evidence that the suppression of K_Ca_3.1 by PGE_2_ is mediated via the EP_2_ receptor.

Because 1-EBIO opens K_Ca_3.1 directly in resting cells by increasing its affinity for Ca^2+^ 19, it suggests that there is tight coupling between the EP_2_ receptor and the channel rather than modulation of intracellular signalling pathways. The ability of PGE_2_ to close K_Ca_3.1 is in keeping with our previous observations that this channel is closed by G_s_-coupled β_2_-adrenoceptors 9 and G_s_-coupled adenosine A_2A_ receptors 10. These effects on K_Ca_3.1 are not mimicked by cAMP analogues or the activator of adenylate cyclase forskolin 9, and considering they are seen in whole-cell configuration of the patch-clamp technique, indicate that the most likely mechanism of action is membrane-delimited involving the G_αs_ or βγ subunits of these GPCR. This view is further supported by the observation that the β_2_-adrenoceptor inverse agonist ICI-118551 actually opens K_Ca_3.1 9, but G_i_ agonists such as CXCL10 and PAF, which lower intracellular cAMP, do not directly activate this channel 6.

The ability of GPCR to modify K_Ca_3.1 appears to be limited to G_s_-coupled receptors as we have not found any evidence that G_i_, G_q_, or G_12/13_-coupled receptors modify K_Ca_3.1 activity. In particular blockade of EP_1_ and EP_2_ in this study did not uncover any G_i_-coupled EP_3_ effects, and GPCR agonists active on human mast cells such as CXCL10 (G_αi_) 6, PAF (G_i_), LPA (G_q, −12/13 and i_), and UTP (G_αq_ P2Y2 receptor) do not open or close K_Ca_3.1 in HLMC. It is well established using cell attached, inside-out and outside patch-clamp recording that most classes of GPCR including G_αs_ couple directly to ion channels through membrane-delimited mechanisms 21, 22. This may lead to either channel opening or channel closing depending on the channel and receptor, and may utilise either the G_α_ component or specific combinations of βγ subunits 21, 22. A further level of specificity between receptor and channel is likely to be achieved by the close approximation of these proteins in tight membrane-restricted signalling complexes. For example, the β_2_ adrenoceptor that can modify both K_Ca_1.1 and voltage-gated Ca^2+^ channel gating is associated with these two channels in a macromolecular complex held together by A-kinase-anchoring proteins 23. It is therefore interesting to speculate that K_Ca_3.1 also localises with the various G_αs_ receptors that modify its function. This and the exact mechanism by which G_αs_ modifies K_Ca_3.1 function will be an important area for future research.

Several molecules that attenuate HLMC secretion including PGE_2_, adenosine and β_2_-adrenoceptor agonists increase intracellular cAMP 24. The generally held view is that this increase in intracellular cAMP couples to inhibition of secretion, supported by the observation that cAMP analogues and non-specific inhibitors of adenylate cyclase can also attenuate secretion from HLMC 24. However, no mechanism has been identified in mast cells that explains how increases in cAMP inhibit the secretory pathway, and the exclusive role of cAMP in the inhibition of other systems such as smooth muscle relaxation has been challenged 25. Opening of K_Ca_3.1 enhances IgE-dependent Ca^2+^ influx and degranulation to a submaximal stimulus 8, and its blockade by charybdotoxin attenuates this 7. Thus, while cAMP plays some role in the inhibition of mast cell mediator release, the demonstration that PGE_2_ also closes K_Ca_3.1 supports the view that cAMP-independent, K_Ca_3.1-dependent mechanisms also contribute in part to EP_2_-dependent inhibition of HLMC mediator release.

In many diseases the recruitment of mast cells to key tissue structures appears critical for their pathophysiological effects 4, 26, 27. For example, the contribution of mast cells to the disordered airway physiology of asthma is undoubtedly facilitated by their migration into the airway epithelium 3, submucosal glands 5 and ASM 4. Inhibition of their migration and subsequent microlocalisation within these structures might therefore offer a novel approach to therapy. Blockade of K_Ca_3.1 markedly inhibits HLMC migration in response to a number of diverse chemotactic stimuli including conditioned medium from activated asthmatic ASM 6. The ability of PGE_2_ to close K_Ca_3.1 suggested that it should also inhibit HLMC migration. However, its ability to inhibit HLMC migration in response to ASM-conditioned medium was variable and did not reach statistical significance. In contrast, the selective EP_2_ receptor agonist butaprost, which was more potent at closing K_Ca_3.1 than PGE_2_, produced a marked and consistent inhibition of HLMC migration. PGE_2_ was also chemotactic at high concentrations when used alone, but was more potent as a chemoattractant when EP_2_ receptors were blocked. This indicates that there are competing pro-migratory and anti-migratory signals when PGE_2_ is present, mediated by the EP_2_ receptor (inhibitory) and most probably the EP_3_ receptor (pro-migratory). These findings are in marked contrast to those in mouse bone marrow-derived mast cells in which PGE_2_ in isolation is a potent chemoattractant. This chemotactic activity is mediated through the EP_3_ receptor, and the lack of inhibition can be attributed to the absence of EP_2_ receptors in mouse bone marrow-derived mast cells 13. In human cord blood-derived mast cells, which express both EP_2_ and EP_3_ receptors, PGE_2_ is not a chemoattractant 13, compatible with an inhibitory role for the EP_2_ receptor. In human lung therefore, PGE_2_ is not likely to act as a HLMC chemoattractant, in keeping with its anti-inflammatory activities in this tissue 18.

In humans PGE_2_ inhibits the early and late asthmatic airway response to allergen challenge 18. The ability of PGE_2_ to close K_Ca_3.1 provides a mechanism through which it is able to achieve these effects. It will be of great interest to investigate whether PGE_2_ has similar effects on lung T-cell K_Ca_3.1 function, cytokine secretion and migration, and whether it can inhibit K_Ca_3.1-dependent ASM proliferation 28. Drugs that block K_Ca_3.1 are also in development as anti-inflammatory treatments 29. Our demonstration that the EP_2_ receptor closes K_Ca_3.1 in HLMC provides further support for the development of specific EP_2_ receptor agonists for the treatment of mast cell-mediated pulmonary disease.

## Materials and methods

### Reagents

We used the following reagents: SCF, IL-6 and IL-10 (R&D, Abingdon, UK); goat polyclonal anti-human IgE, PGE_2_, AH6809, butaprost, PAF, LPA, UTP (Sigma, Poole, Dorset, UK); 17-phenyl trinor PGE_2_ (Cayman Chemical Company, Ann Arbor, Michigan, US); mouse IgG_1_ mAb YB5.B8 (anti-CD117) (Cambridge Bioscience, Cambridge, UK); sheep anti-mouse IgG_1_ Dynabeads (Dynal, Wirral, UK); Dulbecco's Modified Essential Medium (DMEM)/glutamax/HEPES, antibiotic/antimycotic solution, MEM non-essential aminoacids, and fetal calf serum (Life Technologies, Paisley, Scotland, UK).

### Human mast cell purification and culture

All human subjects gave written informed consent, and the study was approved by the Leicestershire Research Ethics Committee. HLMC were dispersed and purified from macroscopically normal lung (*n*=14 donors) obtained within 1 h of resection for lung cancer using immunomagnetic affinity selection as described previously 30. Final mast cell purity determined by Kimura stain was >99% and viability determined by Trypan blue was >97%. HLMC were cultured in DMEM/glutamax/HEPES containing antibiotic/antimycotic solution, non-essential amino acids, 10% fetal calf serum, 100 ng/mL SCF, 50 ng/mL IL-6, and 10 ng/mL IL-10 for up to 10 wk as described previously 8, 31.

### Electrophysiology

The whole-cell variant of the patch-clamp technique was used 7, 32. Patch pipettes were made from borosilicate fibre-containing glass (Clark Electromedical Instruments, Reading, UK), and their tips were heat polished, typically resulting in resistances of 4–6 MΩ. The standard pipette solution contained (in mM) KCl, 140; MgCl_2_, 2; HEPES, 10; Na^+^-ATP, 2; GTP, 0.1 (pH 7.3). The standard external solution contained (in mM) NaCl, 140; KCl, 5, CaCl_2_, 2; MgCl_2_,1; HEPES, 10 (pH 7.3). For recording, mast cells were placed in 35-mm dishes containing standard external solution. Whole-cell currents were recorded using an Axoclamp 200A amplifier (Axon Instruments, Foster City, CA, USA), and currents were evoked by applying voltage commands to a range of potentials in 10 mV steps from a holding potential of −20 mV. The currents were digitised (sampled at a frequency of 10 kHz), stored on computer, and subsequently analysed using pClamp software (Axon Instruments). Capacitance transients were minimised using the capacitance neutralisation circuits on the amplifier. Correction for series resistance was not routinely applied. Experiments were performed at 27°C, and the temperature controlled by a Peltier device. Experiments were performed with a perfusion system (Automate Scientific, San Francisco, CA) to allow solution changes, although drugs were added directly to the recording chamber.

PGE_2_ was dissolved in ethanol to give a stock solution of 10^−2^ M. Thus, at the maximal concentration PGE_2_ used (10^−5^ M), the concentration of ethanol in the recording chamber was 0.1%. This concentration of ethanol did not affect K_Ca_3.1 currents when tested in isolation.

### HLMC chemotaxis

HLMC chemotaxis assays were performed using the Transwell system (BD Biosciences, Oxford, UK) with 24-well plates as described previously 6, 20. Conditioned medium from asthmatic ASM that had been activated with TNFα, IL-1β and IFNγ was placed in the lower wells as described previously 20, with appropriate cytokine containing medium in the negative control. PGE_2_ was added to the bottom wells in the concentration range 10^−6^–10^−3^ M. A total of 1×10^5^ HLMC in 100 µL were added to the top well. After incubating the cells for 3 h at 37°C, we counted the number of HLMC in the bottom well using Kimura stain in a haemocytometer. HLMC migration was calculated as the fold increase of migrated cells in the test wells compared with the negative control (no chemoattractant in the lower well) as described previously 6, 20.

### Data presentation and statistical analysis

Data are expressed as mean±SEM. unless otherwise stated. Differences between groups of data were explored using Student's paired or unpaired *t*-test (two-tailed) as appropriate.

## Abbreviations

ASM: airway smooth muscle
EP: E prostanoid receptor
1-EBIO: 1-ethyl-2-benzimidazolinone
GPCR: G protein-coupled receptor
HLMC: human lung mast cell
LPA: lysophosphatidic acid
PAF: platelet activating factor
PG: prostaglandin
